# A Standard Herbal Formula, CGAC, Attenuates Bone Loss by Normalizing Low-Bone Turnover Stagnation in an Orchiectomy-Induced Mouse Model

**DOI:** 10.3390/ph19040555

**Published:** 2026-03-31

**Authors:** Dong-Cheol Baek, Min-Young Chae, Tae-Wook Woo, Chang-Gue Son, Eun-Jung Lee

**Affiliations:** 1 Institute of Bioscience & Integrative Medicine, Daejeon University, Daejeon 35235, Republic of Korea; berk9@naver.com (D.-C.B.); apfhs98@naver.com (T.-W.W.); 2 Department of Korean Rehabilitation Medicine, College of Korean Medicine, Daejeon University, Daejeon 35235, Republic of Korea; cdy6401@naver.com

**Keywords:** CGAC, male osteoporosis, orchiectomy, bone mineral density, bone remodeling

## Abstract

**Background/Objectives**: Osteoporosis is a progressive systemic skeletal disease, with male osteoporosis emerging as a critical global concern due to high morbidity and mortality from fractures. This study investigated the anti-osteoporotic potential of CGAC—a herbal mixture of *Cervus elaphus Linnaeus*, *Glycine max* (L.) Merr., *Angelica gigas* Nakai, and *Cnidium officinale* Makino—and its underlying mechanisms in an orchiectomized (ORX) mouse model. **Methods**: C57BL/6J mice underwent ORX for 8 weeks, followed by CGAC administration (250 and 500 mg/kg) for an additional 8 weeks. Molecular mechanisms were further validated using MG63 osteoblastic and RAW 264.7 osteoclast assays. **Results**: ORX induced severe osteoporotic phenotypes, including significant reductions in bone mineral density (BMD) and trabecular microarchitecture. Notably, at the time point examined, ORX was associated with a suppressed bone remodeling state, reflected by reductions in both TRAP-positive osteoclasts and ALP-positive osteoblasts, together with lower serum BALP, CTX-1, and Gla/Glu-OC ratio. Conversely, CGAC normalized this stagnant state and restored physiological remodeling. This was accompanied by reduced marrow fat accumulation through the AMPK signaling axis, which upregulated Runx2 and downregulated PPAR-γ. In vitro results confirmed that CGAC promoted osteoblast differentiation and mineralization while suppressing RANKL-induced osteoclastogenesis. These actions suggest that CGAC may be involved in regulating Wnt/β-catenin signaling. **Conclusions**: Overall, CGAC is a promising therapeutic candidate for male osteoporosis, offering pharmacological benefits particularly relevant to aging populations.

## 1. Introduction

Osteoporosis is a major systemic skeletal disease characterized by low bone density and abnormal microarchitecture [1]. Although it is often considered a disease that primarily affects postmenopausal women, the burden of male osteoporosis has grown rapidly with global population aging [2]. Men constitute approximately 20% of all osteoporosis cases, and their one-year mortality post-fragility fractures approaches 20%, nearly twice that of women [3]. These data demonstrate the need for treatments specific to the biological characteristics of male osteoporosis.

A reduction in androgen levels is a primary factor contributing to male bone loss [4]. Androgens, especially testosterone, contribute to male skeletal homeostasis through both direct androgen receptor-mediated effects and indirect estrogenic effects after aromatization to estradiol. In men, estradiol is particularly important for restraining bone resorption, whereas both testosterone and estradiol contribute to maintenance of bone formation and bone mass [4,5]. When androgen levels decrease, the differentiation of osteoblasts is inhibited, the Wnt/β-catenin and IGF-1 signaling pathways are blocked, marrow adipogenesis accelerates, and RANKL-mediated osteoclastogenesis is elevated [6]. Testosterone levels naturally decrease at a rate of about 1% per year, and this decline contributes to bone fragility [7]. In a clinical study of 2639 men aged over 65, testosterone levels showed a positive association with BMD and were inversely related to the risk of fractures in the hip, femur, and arm [8].

Current pharmacologic treatments such as bisphosphonates and denosumab are recommended first-line therapies that reduce fracture risk by suppressing osteoclast-driven bone resorption; however, they have a few side effects [9,10]. Long-term bisphosphonate use often requires drug holidays due to safety concerns [11], and denosumab withdrawal can trigger reverse bone loss and increase fracture risk [12]. Importantly, these drugs act primarily through anti-resorptive pathways and are not direct osteoanabolic agents, although they may exert indirect structure-preserving or remodeling-stabilizing effects by reducing excessive resorption and permitting secondary mineralization. Therefore, despite the availability of effective agents, there remains a need for therapeutic candidates that can simultaneously influence multiple disrupted processes in androgen-deficient bone, such as impaired osteoblastogenesis, excessive marrow adiposity, and abnormal remodeling balance. In this context, a multi-component herbal formulation such as CGAC may offer pharmacological relevance beyond a single-pathway intervention.

CGAC is a multi-herbal formulation composed of *Cervus elaphus Linnaeus*, *Glycine max* (L.) Merr., *Angelica gigas* Nakai, and *Cnidium officinale* Makino. Each component has been traditionally used to strengthen bones, tonify and generate marrow and remove blood stasis, and experimental evidence supports their individual osteogenic or anti-osteoclastic actions [13,14,15,16]. *Cervus elaphus sibiricus* and *Glycine max* (L.) Merr regulate osteogenic molecules [17], *Angelica gigas* Nakai promotes bone formation [18], and *Cnidium officinale* Makino inhibits osteoclastogenesis in ovariectomy-induced osteoporotic models [19]. However, the combined effects of these herbs have not been evaluated in male or androgen-deficient osteoporosis. Given their complementary mechanisms, CGAC presents a biological rationale as a multi-target therapeutic candidate.

Herein, we aimed to investigate the anti-osteoporotic effects of CGAC and to examine its potential mechanisms in an orchiectomized (ORX) mouse model of testosterone-deficient osteoporosis. We predefined femoral bone mineral density (BMD) and trabecular microarchitectural parameters as the primary endpoint because preservation of bone mass is the most clinically relevant indicator of anti-osteoporotic efficacy in this model. Secondary endpoints included serum bone turnover markers, histomorphometric indices, and in vitro osteoblast/osteoclast functional assays. Our null hypothesis was that CGAC would not differ from untreated ORX controls in BMD or bone remodeling-related vvoutcomes.

## 2. Results

### 2.1. Fingerprinting of CGAC

**Figure 1:**
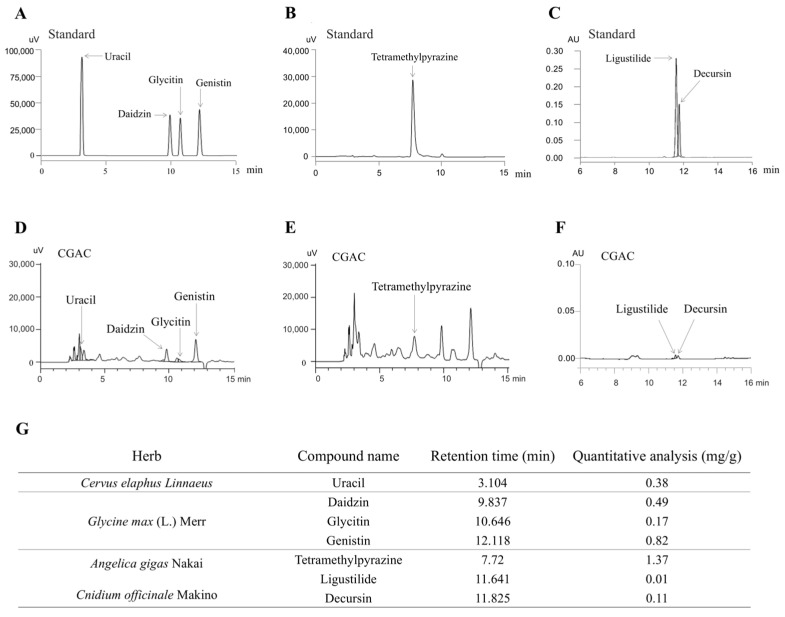
Fingerprinting analysis of CGAC. Chemical compositions and quantitative analysis of seven reference standards (**A**–**C**) and CGAC (**D**–**F**) using high-performance liquid chromatography (HPLC). A quantitative analysis of CGAC was conducted (**G**).

Seven compounds—uracil, daidzin, glycitin, genistin, tetramethylpyrazine, ligustilide, and decursin—were detected at retention times of 3.104, 9.837, 10.646, 12.118, 7.72, 11.641, and 11.825 min, respectively, in CGAC. Semiquantitative analysis showed 0.38 mg/g uracil, 0.49 mg/g daidzin, 0.17 mg/g glycitin, 0.82 mg/g genistin, 1.37 mg/g tetramethylpyrazine, 0.01 mg/g ligustilide, and 0.11 mg/g decursin in CGAC (Figure 1).

### 2.2. CGAC Attenuated ORX-Induced Bone Loss

**Figure 2:**
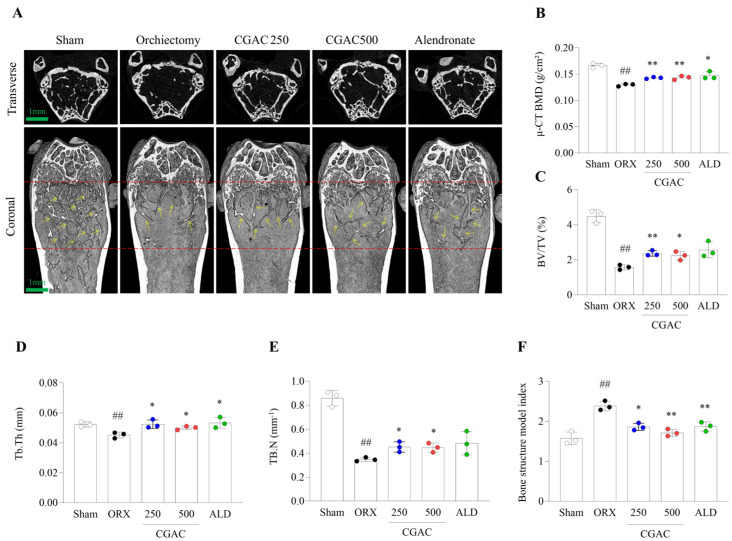
Inhibitory effects of CGAC on ORX-induced bone loss. Representative µCT images of the femur (scale bar: 1 mm) (**A**). The scores of bone mineral density (BMD) (**B**), bone volume/total volume (BV/TV) (**C**), trabecular bone thickness (Tb. Th) (**D**), trabecular bone number (Tb. N) (**E**), and structure model index (SMI) were analyzed by µCT (**F**). White dot: sham group; black dot: orchiectomy group; blue dot: orchiectomy + CGAC 250 mg/kg treatment group; red dot: orchiectomy + CGAC 500 mg/kg treatment; green dot: sham group. yellow arrow: trabecular bone formation. The data are presented as the mean ± SD. ## *p* < 0.01, compared to the sham group; * *p* < 0.05, ** *p* < 0.01, compared to the ORX group. Mean differences and 95% confidence intervals for the principal comparisons are provided in Supplementary Table S1. Sham, sham-operated mice that underwent the same surgical procedure without orchiectomy; ORX, orchiectomy; CGAC 250, CGAC 250 mg/kg; CGAC 500, CGAC 500 mg/kg; ALD, alendronate.

µCT images clearly demonstrated the onset of osteoporosis in ORX-induced mice (Figure 2A). This observation was further supported by decreases in µCT-BMD, along with reductions in trabecular bone parameters such as BV/TV, Tb, Th, Tb. N, and SMI. Notably, the administration of CGAC significantly mitigated these declines compared to the ORX group (Figure 2B–F).

**Figure 3:**
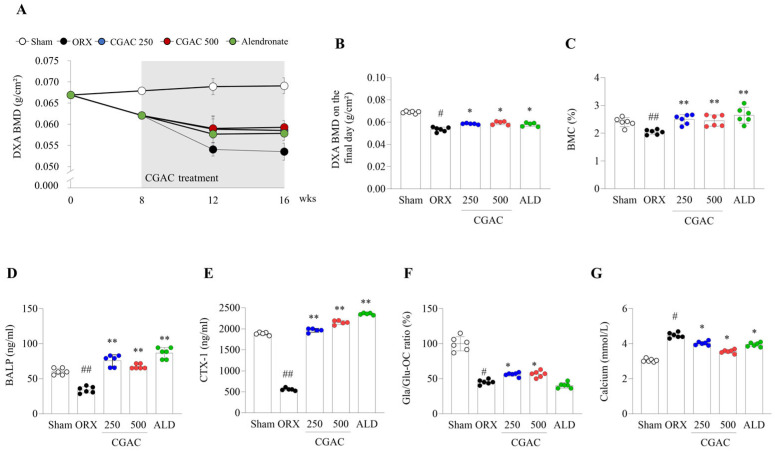
Effects of CGAC on BMD and serum bone turnover markers in ORX mice. Changes in BMD over 16 weeks (**A**), BMD at 16 weeks (**B**), and BMC (**C**) measured via dual-energy X-ray absorptiometry (DXA). Levels of bone-specific alkaline phosphatase (BALP) (**D**), telopeptide of collagen type 1 (CTX-1) (**E**), the ratio of carboxylated to uncarboxylated osteocalcin (Gla/Glu-OC) (**F**), and calcium (**G**) in serum were analyzed by ELISA. White dot: sham group; black dot: orchiectomy group; blue dot: orchiectomy + CGAC 250 mg/kg treatment group; red dot: orchiectomy + CGAC 500 mg/kg treatment; green dot: sham group. The data are presented as the mean ± SD. # *p* < 0.05, ## *p* < 0.01, compared to the sham group; * *p* < 0.05, ** *p* < 0.01, compared to the ORX group. Mean differences and 95% confidence intervals for the principal comparisons are provided in Supplementary Table S2. Sham, sham-operated mice that underwent the same surgical procedure without orchiectomy; ORX, orchiectomy; CGAC 250, CGAC 250 mg/kg; CGAC 500, CGAC 500 mg/kg; ALD, alendronate.

DXA-BMD levels decreased by 9%, 19%, and 22% at 8, 12, and 16 weeks after ORX, respectively, compared with those in the sham group. CGAC administration markedly attenuated this loss, improving BMD by approximately 12% and increasing BMC by 16% relative to the ORX group (Figure 3A–C).

### 2.3. CGAC Normalized Bone-Turnover Markers

ORX notably lowered the serum levels of BALP, CTX-1 and Gla/Glu-OC ratios and increased Ca compared with those in the sham group. However, the administration of CGAC significantly increased the levels of BALP (*p* < 0.01), CTX-1 (*p* < 0.01) and the Gla/Glu-OC ratio (*p* < 0.05), and attenuated Ca (*p* < 0.05) (Figure 3D–G).

### 2.4. CGAC Attenuated Fat Accumulation and Its Related Molecules in the Femur

**Figure 4:**
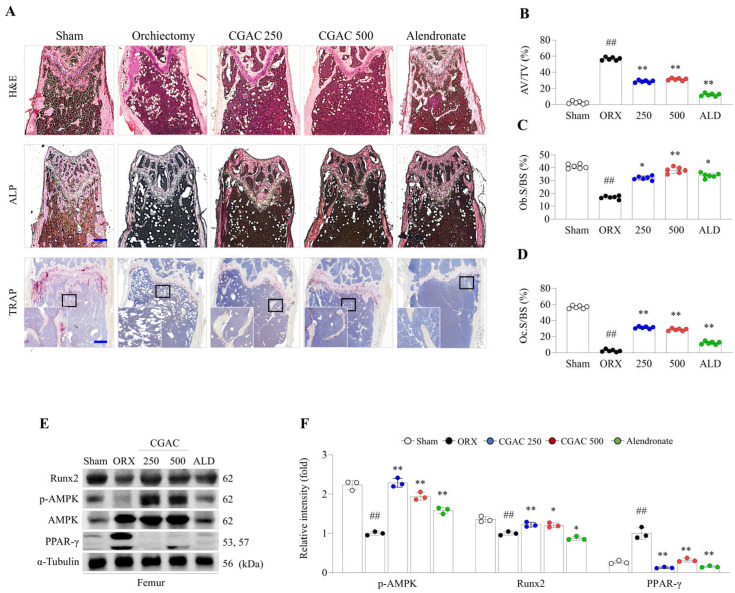
CGAC decreased fat accumulation and increased bone formation in the femur. Evaluation of histological alterations using H&E, ALP, and TRAP staining (**A**) was performed, and representative photographs (40× magnification) were semi-quantified for bone marrow adipocyte volume/tissue volume (AV/TV) (**B**), osteoblast surfaces/bone surface (Ob.S/BS) (**C**), and osteoclast surfaces/bone surface (Oc.S/BS) (**D**) at the secondary spongiosa in the femur of ORX-induced mice. Fat accumulation- and bone formation-related proteins were visualized by Western blot in the femur (**E**), and ImageJ (version 1.46) was used to semi-quantify the relative intensities of the band (**F**). White dot: sham group; black dot: orchiectomy group; blue dot: orchiectomy + CGAC 250 mg/kg treatment group; red dot: orchiectomy + CGAC 500 mg/kg treatment; green dot: sham group. The data are presented as the mean ± SD. ## *p* < 0.01, compared to the sham group; * *p* < 0.05, ** *p* < 0.01, compared to the ORX group. Mean differences and 95% confidence intervals for the principal comparisons are provided in Supplementary Table S3. Sham, sham-operated mice that underwent the same surgical procedure without orchiectomy; ORX, orchiectomy; CGAC 250, CGAC 250 mg/kg; CGAC 500, CGAC 500 mg/kg; ALD, alendronate.

ORX increased bone marrow adipocyte volume in the secondary spongiosa of the femur, which was accompanied by a reduction in both Ob.S/BS and trabecular bone area by H&E and ALP staining. However, CGAC administration remarkably attenuated these alterations (Figure 4A–D) and related protein expression, notably increasing Runx2 and p-AMPK and suppressing PPAR-γ in the femur (Figure 4E,F). However, TRAP staining showed that Oc.S/BS was significantly lower in the ORX group than in the sham group in the trabecular bone of the femur. Notably, CGAC administration exhibited increased osteoclast activity, maintaining levels comparable to the sham group, which were significantly higher than those observed in the ORX group (Figure 4A,D).

### 2.5. CGAC Promoted Osteoblastic Bone Formation and Inhibited Osteoclast Activity

**Figure 5:**
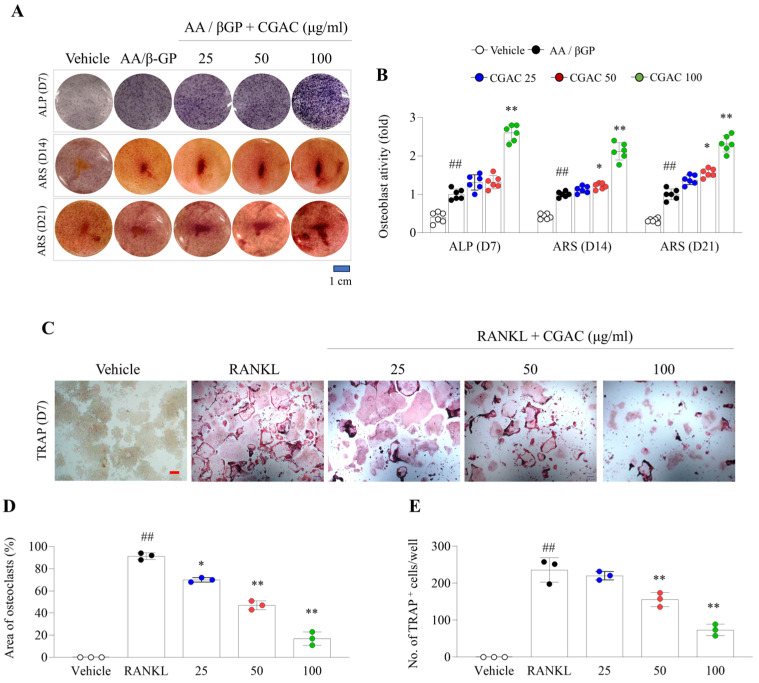
CGAC promoted bone formation and inhibited bone resorption in vitro. In MG63 cells, ALP staining and ARS staining plate image at 7, 14, and 21 days, respectively (**A**), and staining intensity was quantified by absorbance at 405 and 550 nm (**B**). In RAW 264.7 cells, TRAP staining was performed after 7 days of RANKL-induced differentiation (Scale bar: 200 μm) (**C**), and the TRAP-positive area (**D**) and number of TRAP-positive multinucleated cells (**E**) were quantified using software (version 1.46). White dot: vehicle group; black dot: RANKL group; blue dot: RANKL + CGAC 25 μg/mL treatment group; red dot: RANKL + CGAC 50 μg/mL treatment; green dot: RANKL + CGAC 100 μg/mL treatment. The data are presented as the mean ± SD. ## *p* < 0.01, compared to the vehicle group; * *p* < 0.05, ** *p* < 0.01, compared to the AA/βGP group or RANKL group. Mean differences and 95% confidence intervals for the principal comparisons are provided in Supplementary Table S4. AA/βGP, ascorbic acid/β-glycerophosphate; CGAC 25, CGAC 25 μg/mL; CGAC 50, CGAC 50 μg/mL; CGAC 100, CGAC 100 μg/mL; ALD, alendronate.

CGAC treatment notably promoted osteogenic differentiation of MG63 cells, as evidenced by increased ALP activity and calcium deposition under AA/βGP-induced differentiation conditions (Figure 5A,B). In addition, CGAC suppressed RANKL-induced osteoclastogenesis in RAW 264.7 cells, as shown by reduced TRAP-positive area and fewer TRAP-positive multinucleated cells in a dose-dependent manner (Figure 5C–E).

### 2.6. CGAC Was Associated with Changes in Wnt/β-Catenin-Related Proteins in MG63 Cells

**Figure 6:**
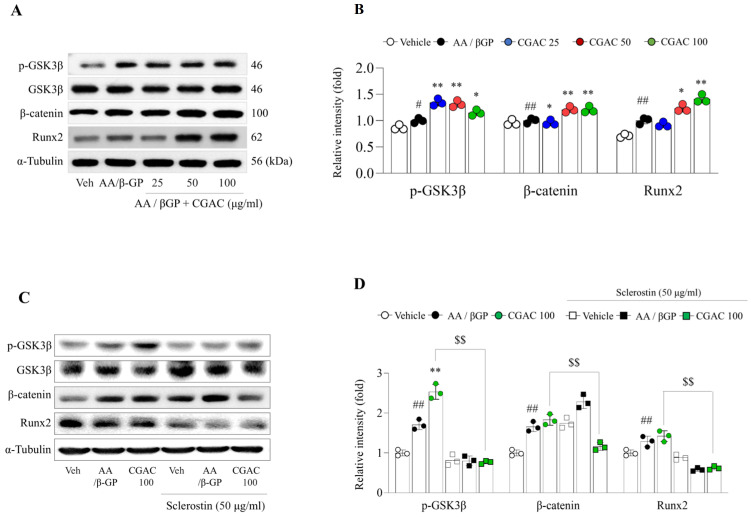
CGAC activated Wnt/β-catenin-related osteogenic signaling in MG63 cells. Protein expression levels of p-GSK3β, β-catenin, and Runx2 were analyzed by Western blot in MG63 cells treated with CGAC under osteogenic differentiation conditions (**A**), and band intensities were semi-quantified using software (version 1.46) (**B**). Sclerostin expression was also evaluated by Western blot (**C**) and semi-quantified using ImageJ (**D**). White circle: vehicle group; black circle: AA/βGP group; green circle: AA/βGP + CGAC 100 μg/mL treatment; White square: vehicle group + sclerostin; black square: AA/βGP group + sclerostin; green square: AA/βGP + CGAC 100 μg/mL treatment + sclerostin. The data are presented as the mean ± SD. # *p* < 0.05, ## *p* < 0.01, compared to the vehicle group; * *p* < 0.05, ** *p* < 0.01, compared to the AA/βGP group; $$ *p* < 0.01, compared to the AA/βGP + CGAC 100 group; Mean differences and 95% confidence intervals for the principal comparisons are provided in Supplementary Table S5. AA/βGP, ascorbic acid/β-glycerophosphate; CGAC 25, CGAC 25 μg/mL; CGAC 50, CGAC 50 μg/mL; CGAC 100, CGAC 100 μg/mL; ALD, alendronate.

In MG63 cells cultured under AA/βGP-induced osteogenic conditions, CGAC treatment significantly increased the protein expression levels of p-GSK3β (*p* < 0.01 for 25 or 50 μg/mL and *p* < 0.05 for 100 μg/mL), β-catenin (*p* < 0.05 for 25 μg/mL and *p* < 0.01 for 50 or 100 μg/mL), and Runx2 (*p* < 0.05 for 50 μg/mL and *p* < 0.01 for 100 μg/mL) (Figure 6A,B). This effect was markedly attenuated by co-treatment with sclerostin (50 μg/mL). In the presence of sclerostin, the CGAC-induced increases in p-GSK3β, β-catenin, and Runx2 protein expression were significantly suppressed compared with CGAC treatment alone, indicating that activation of the GSK3β/β-catenin/Runx2 signaling pathway by CGAC was inhibited by sclerostin (Figure 6C,D).

## 3. Discussion

Although male osteoporosis imposes a growing global burden, its biological features and therapeutic responses remain incompletely characterized [20]. In this study, we evaluated the anti-osteoporotic potential of CGAC, a multi-component herbal formulation with putative bone-forming activity and investigated its mechanisms in models of androgen-deficient bone loss in vivo and in vitro. As expected, ORX induced significant reductions in BMD together with trabecular microstructural deterioration (Figure 2 and Figure 3), consistent with its established use as a model of male osteoporosis caused by sex steroid deficiency [21].

Testosterone contributes to male skeletal homeostasis through both direct androgen receptor-mediated effects and indirect effects following aromatization to estradiol, thereby supporting bone formation and restraining excessive bone resorption [22,23]. In the present study, CGAC attenuated BMD loss during the treatment period and improved trabecular parameters, including Tb.Th, Tb.N, and SMI, indicating partial restoration of trabecular integrity (Figure 2 and Figure 3). Forty-four androgen deficiency-related male osteoporosis patients present both reduced BMD and low mean values of trabecular bone parameters (Tb.Th., Tb.N., and node count) [24]. In addition to structural deterioration, androgen deficiency disrupts the marrow microenvironment and shifts mesenchymal precursors toward adipogenic rather than osteogenic differentiation [25,26]. Consistent with this concept, CGAC markedly reduced marrow fat accumulation (Figure 4A,B), suggesting improved osteogenic commitment and reduced marrow adiposity.

AMPK appears to play context-dependent roles in bone remodeling by promoting osteoblast differentiation and metabolic fitness in some settings while restraining osteoclastogenesis in others [27]; it restrains osteoclastogenesis and RANKL-related resorptive activity in other contexts [28]. In agreement with previous reports on AMPK activation in bone-related metabolic regulation [29], CGAC significantly increased femoral AMPK expression in ORX mice, accompanied by suppression of PPAR-γ and enhancement of Runx2 (Figure 4D,E). These findings suggest that CGAC may protect against androgen-deficiency-related bone loss, at least in part, by improving the balance between osteogenesis and marrow adipogenesis.

One notable finding was that, by week 16, the ORX model exhibited features consistent with a low-turnover-like remodeling state. Histological staining showed reductions in both TRAP- (Figure 4A,D) and ALP-positive areas (Figure 4A,C), and these changes were paralleled by lower serum BALP, CTX-1, and the Gla/Glu-OC ratio in the ORX group (Figure 3D–F). Such a synchronized decline in both histological and serum markers mirrors the clinical low-turnover state observed in men with severe, long-term hypogonadism [30]. Although androgen deficiency is often associated with increased turnover in earlier phases of bone loss, our results suggest that, at this later point, severe trabecular depletion may have been accompanied by suppressed remodeling activity. In this context, the higher cellular activity observed in the sham group should be interpreted as physiological age-related remodeling rather than pathological destruction (Figure 4A; TRAP staining). Consistent with our data, a similar pattern was reported in the senescence-accelerated mouse (SAMP8) model. Although SAMR1 control mice exhibited significantly higher BMD than the osteoporotic SAMP8 mice, they also demonstrated greater TRAP-positive areas and elevated serum TRACP-5b levels [31]. Therefore, CGAC maintained histological and biochemical indices closer to the sham group, suggesting preservation of remodeling capacity through protection of skeletal microarchitecture.

The in vitro findings were broadly consistent with the in vivo data. CGAC promoted osteogenic differentiation and mineralized nodule formation in MG63 cells while suppressing RANKL-induced osteoclastogenesis in RAW264.7 cells (Figure 5). The fact that the in vitro results—demonstrating both pro-anabolic and anti-resorptive properties—match the in vivo histological staining patterns and serum markers (BALP, Gla/Glu-OC ratio, calcium) further reinforces the multi-targeted efficacy of the formulation. The in vitro outcomes suggest that CGAC modulates both osteoblast and osteoclast lineages in a coordinated manner rather than acting on a single cellular process.

Several signaling pathways—including BMP/SMAD, IGF-1/PI3K-AKT, and Wnt/β-catenin—are known to regulate osteoblast differentiation and bone matrix formation in a coordinated manner [32]. Among the pathways implicated in osteoblast differentiation, Wnt/β-catenin signaling is a central regulator of osteoblast commitment and trabecular rather than acting through a single molecular target [33,34,35]. HPLC fingerprinting identified representative marker compounds derived from the four constituent materials, supporting extract standardization and suggesting that the biological effects of CGAC may arise from the combined actions of multiple osteogenic, angiogenic, and anti-inflammatory constituents, including uracil (from *Cervus elaphus Linnaeus*), daidzin, glycitin, and genistin (from *Glycine max* (L.) Merr), decursin and ligustilide (from *Angelica gigas* Nakai), and tetramethylpyrazine (from *Cnidium officinale* Makino) (Figure 1). These molecules have been reported to possess osteogenic [36], angiogenic [37], or anti-inflammatory properties [38], which could collectively underline the observed anti-osteoporotic effects of CGAC. In addition, preliminary safety observations indicated that CGAC was well tolerated under the tested conditions, as a 4-week administration at 1 g/kg did not increase serum BUN, AST, or ALT levels (Figure S1). These findings indicate that CGAC is well tolerated at the tested dose and support its potential as a safe, natural anabolic therapeutic candidate for male osteoporosis.

However, this study has several limitations that should be considered when interpreting the findings. First, although alendronate was selected as a clinically relevant first-line anti-resorptive comparator, we did not directly compare CGAC with other therapeutic classes such as teriparatide, SERMs, or romosozumab. Second, although CGAC was associated with changes in AMPK- and Wnt/β-catenin-related markers, direct pathway validation was not performed, and the primary molecular targets remain to be defined, including nuclear β-catenin localization and TCF/LEF transcriptional assays. Third, the present study was not designed to determine which constituents are the principal bioactive compounds or to define their individual contributions to the observed pharmacological effects. Further fractionation and constituent-level studies are needed to identify the principal active components. Finally, the use of MG63 cells limits physiological interpretation because these osteosarcoma-derived cells do not fully recapitulate normal osteoblast biology. Further comparative, mechanistic, and primary-cell studies will be needed to clarify the translational potential of CGAC.

## 4. Materials and Methods

### 4.1. Chemicals and Reagents

The following reagents and chemicals were obtained from commercial suppliers: 10% neutral buffered formalin, tetraethyl ethylenediamine (TEMED), aqueous mounting solution, acetic acid, BCA protein assay kit, Decalcifying Solution-Lite, N-(1-naphthyl)-ethylenediamine dihydrochloride, and Tween 20 were purchased from Sigma-Aldrich (St. Louis, MO, USA). Antibiotic-antimycotic mixture, trypsin–EDTA, Dulbecco’s Modified Eagle Medium (DMEM), Dulbecco’s Phosphate-Buffered Saline (DPBS), and fetal bovine serum (FBS) were acquired from Welgene (Daegu, Republic of Korea). Bovine serum albumin (BSA) was sourced from GenDEPOT (Barker, TX, USA). Protease and phosphatase inhibitors, along with RNA Later, were obtained from Thermo Fisher Scientific (Waltham, MA, USA). Skim milk powder, RIPA lysis buffer, and 10% ammonium persulfate were supplied by LPS Solution (Daejeon, Republic of Korea). PVDF membranes were purchased from Pall Corporation (Port Washington, NY, USA), and methylene alcohol was obtained from Daejung Chemicals & Metals Co. (Siheung, Republic of Korea). Mayer’s hematoxylin was sourced from Wako Pure Chemical Industries (Osaka, Japan). Proprep™ protein extraction solution was obtained from iNtRON Biotechnology (Seongnam, Republic of Korea). Antibodies against α-tubulin and β-catenin were obtained from Abcam (Cambridge, MA, USA), while those targeting Runx2, p-AMPKα, AMPKα, p-GSK3β, GSK3β, and PPAR-γ were procured from Cell Signaling Technology (Danvers, MA, USA).

### 4.2. CGAC Preparation

*Cervus elaphus Linnaeus* (Cervi Cornu; Nokgak, code: HCDR-220922), i.e., the fully ossified antler, *Glycine max* (L.) Merr. (code: 230924-299), *Angelica gigas* Nakai (code: 220704-234), and *Cnidium officinale* Makino (code: 230607-46) were purchased from Jeong-Seong Korean-Drugstore (Daejeon, Republic of Korea). A total of 100 g of the mixed raw materials was used for extraction, consisting of *Cervus elaphus Linnaeus* (24 g), *Glycine max* (L.) Merr. (36 g), *Angelica gigas* Nakai (20 g), and *Cnidium officinale* Makino (20 g), corresponding to a 24:36:20:20 weight ratio. The powdered mixture was added to 1 L of distilled water and extracted at 100 °C for 3 h using a reflux extraction system. The extract was cooled to room temperature (25 °C) and filtered twice through Whatman No. 2 filter paper (Maidstone, UK) to remove insoluble residues. The filtrate was concentrated under reduced pressure at 60 °C using a rotary evaporator (Rotavapor R-300, Büchi, Switzerland) and subsequently lyophilized for 48 h using a freeze dryer to yield a fine brown powder. The final dry extract was designated as CGAC, with a total yield of 5.6% (*w*/*w*) based on the starting material weight. The lyophilized powder was stored at −20 °C until further use in vivo and in vitro experiments.

### 4.3. Fingerprinting of CGAC

HPLC fingerprinting was conducted to characterize the chemical composition of CGAC and to identify its principal bioactive constituents. Based on previous literature, CGAC contains several compounds known to exhibit osteogenic or anti-inflammatory activity, including uracil (from *Cervus elaphus Linnaeus;* Nokgak), isoflavones such as daidzin, glycitin, and genistin (from *Glycine max* (L.) Merr.), and tetramethylpyrazine, ligustilide, and decursin (from *Angelica gigas* Nakai and *Cnidium officinale* Makino). For chromatographic analysis, 100 mg of CGAC powder and analytical standards of uracil (8 mg), daidzin (16 mg), glycitin (16 mg), genistin (16 mg), tetramethylpyrazine (5 mg), decursin (5 mg), and ligustilide (5 mg) were each dissolved in 50 mL of 50% methanol (*v*/*v*). The solutions were sonicated for 15 min at 25 °C and filtered through 0.45 μm PVDF membrane filters (Millipore, Billerica, MA, USA) before injection. Chromatographic separation was performed using a Waters Alliance e2695 HPLC system equipped with a PDA detector (Waters 2998, Milford, MA, USA). The analytical column was a SunFire C18 column (5 μm, 4.6 × 250 mm; Waters, USA) maintained at 30 °C. The mobile phase consisted of: Solvent A: 0.05% phosphate in distilled water; and Solvent B: acetonitrile containing 0.05% phosphate. The gradient elution program was as follows: 0–10 min, 10% B; 10–30 min, 10–40% B; 30–45 min, 40–70% B; 45–50 min, 70–90% B; followed by re-equilibration to 10% B for 10 min. The flow rate was maintained at 1.0 mL/min, and the injection volume was 20 μL. Detection was performed at 240–450 nm with a spectral acquisition rate of 1 Hz. Data were processed using Empower 3.0 software (Waters, USA). Each compound in CGAC was identified by comparing its retention time and UV spectrum with those of corresponding reference standards.

### 4.4. Animals and Orchiectomy

Forty male C57BL/6J mice (11 weeks old, 25–27 g) were obtained from Dae Han Bio Link (Eumseong, Republic of Korea). Animals were housed under controlled environmental conditions (temperature 22 ± 1 °C, relative humidity 60 ± 4%, 12 h light/dark cycle) with free access to standard rodent chow and tap water. All experimental procedures were approved by the Institutional Animal Care and Use Committee of Daejeon University (Approval No. DJUARB2023-039, Daejeon, Republic of Korea) and conducted in strict accordance with the ARRIVE guidelines 2.0 for the care and use of laboratory animals. Mice were acclimated for one week prior to the start of the experiment.

Orchiectomy (ORX) Procedure [39]: At 12 weeks of age, mice were anesthetized via intraperitoneal injection of ketamine (90 mg/kg) and xylazine (10 mg/kg). After shaving and disinfecting the scrotal area with povidone-iodine, a small longitudinal incision was made in the scrotal skin, and the underlying connective tissues were gently separated. The testicular blood vessels were isolated and ligated using sterile surgical sutures, after which the testis, epididymides, and associated adipose tissues were carefully excised. The incision was closed with absorbable sutures, and the site was again treated with povidone-iodine to minimize postoperative infection. Mice in the sham group underwent the same surgical procedure except for the removal of reproductive tissues. Postoperative care included monitoring until full recovery from anesthesia and daily health observation. At 28 weeks of age, all animals were euthanized by controlled CO_2_ inhalation, and blood and femur samples were collected. Serum was separated by centrifugation and, together with femoral tissues, stored at −80 °C until further biochemical and histological analyses.

### 4.5. Experimental Group and Drug Treatment

Eight weeks after orchiectomy (ORX), when osteoporosis was confirmed by dual-energy X-ray absorptiometry (DXA; InAlyzer, Medikors, Republic of Korea), the mice were randomly assigned to five groups (*n* = 8 per group): Sham—Sham-operated + tap water, ORX—ORX + tap water, CGAC 250—ORX + CGAC 250 mg/kg, CGAC 500—ORX + CGAC 500 mg/kg, ALD—ORX + alendronate (5 mg/kg; Sigma-Aldrich, USA). To estimate clinically relevant in vivo exposure, the traditional human daily dose of the crude herbal materials (approximately 10–30 g/day for a 60-kg adult) was converted using body surface area normalization. Considering the 5.6% extraction yield, the selected mouse doses correspond approximately to human extract-equivalent doses of about 1.2 g/day (250 mg/kg) and 2.4 g/day (500 mg/kg) for a 60 kg adult. Thus, the lower dose was intended to reflect a clinically plausible exposure range, whereas the higher dose was included as an exploratory upper dose to assess dose responsiveness. CGAC powder was freshly dissolved in distilled water prior to administration. All treatments were administered orally once daily for 8 weeks using an oral gavage needle in a dosing volume of 10 mL/kg body weight. Body weight and food intake were monitored weekly throughout the experimental period.

The sample size (*n* = 8 per group) was determined based on previous ORX mouse studies and practical/ethical considerations for animal experimentation. This group size was considered sufficient to detect biologically meaningful differences in the primary endpoint (BMD). To improve transparency, we now state this rationale explicitly.

### 4.6. Micro-Computed Tomography (µCT) Imaging Analysis

To confirm the bone loss induced by ORX, trabecular bone in the femur (secondary spongiosa, 3 mm distal to the growth plate) was analyzed using micro-computed tomography (micro-CT; Skyscan 1172, Kontich, Belgium). 2D and 3D image analyses of femurs were reconstructed in NRecon (Bruker, Belgium) and datasets were reoriented in Data Viewer (Bruker, Belgium). The radiographic projections were acquired at 60 kV and 167 μA, and each of the samples was scanned at a high resolution (5.89 μm/pixel). Morphological measurements for the trabecular bone of the femur included structure model index (SMI), bone mineral density (BMD), trabecular number (Tb.N.), bone volume/total volume (BV/TV) and trabecular thickness (Tb. Th).

### 4.7. Dual Energy X-Ray Absorptiometry Analysis

After euthanasia, femurs were harvested from each mouse and analyzed using dual-energy X-ray absorptiometry (DXA) with an InAlyzer system (Medikors Co., Seongnam, Korea) to quantify bone mineral density (BMD, g/cm^2^) and bone mineral content (BMC, g). For each sample, the entire femur—from the proximal head to the distal condyle—was scanned in the high-resolution mode. Regions of interest (ROIs) were manually delineated along the cortical and trabecular regions of the femur using the manufacturer’s image analysis software (InAlyzer v1.3.0), following the standard analytical protocol provided by Medikors. Each femur was scanned five times independently, and the mean value of the five measurements was recorded as the representative BMD and BMC for each mouse. All DXA measurements were performed by a single blinded examiner (M.-Y.C) under identical calibration and exposure settings to ensure reproducibility.

### 4.8. Histological Analysis

Femurs were fixed in 10% neutral-buffered formalin for 24 h at room temperature, followed by decalcification in a commercial decalcifying solution (24% formic acid and 0.5% formalin; Wako Pure Chemical, Japan) at 4 °C for four weeks with solution changes every 3–4 days. After decalcification, the specimens were rinsed in running tap water for 1 h, dehydrated through a graded ethanol series, cleared in xylene, and embedded in paraffin. Serial sagittal sections of the distal femur were cut at a thickness of 8 μm using a rotary microtome (Leica RM2235, Nussloch, Germany). For general histological assessment, sections were stained with Mayer’s hematoxylin and eosin (H&E) following standard protocols. Alkaline phosphatase (ALP) staining kit and a TRAP (tartrate-resistant acid phosphatase) staining kit (Takara Bio Inc., Shiga, Japan) were used according to the manufacturer’s instructions. Stained sections were examined and imaged under an Axiophot microscope (Carl Zeiss, Jena, Germany) equipped with a digital camera (AxioCam HRc, Carl Zeiss). Quantitative morphometric analysis was performed on the secondary spongiosa region, located 200–400 μm below the distal growth plate. Parameters including adipocyte volume relative to bone volume (AV/BV, %) and osteoblast surface relative to bone surface (Ob.S/BS, %) were calculated using ImageJ software (NIH, Bethesda, MD, USA). At least three non-overlapping fields per section and three sections per mouse were analyzed to obtain representative values.

### 4.9. Western Blot Analysis

Femoral tissues were pulverized into fine powder under liquid nitrogen using a pre-chilled mortar and pestle. The powdered samples were lysed in RIPA buffer (Thermo Fisher Scientific, Waltham, MA, USA) supplemented with protease and phosphatase inhibitor cocktails (Sigma-Aldrich, St. Louis, MO, USA). The lysates were centrifuged at 12,000× *g* for 15 min at 4 °C, and the supernatants were collected for protein quantification using the BCA Protein Assay Kit (Thermo Fisher Scientific). Equal amounts of total protein were separated on 10% SDS-PAGE gels and electrotransferred onto polyvinylidene fluoride (PVDF) membranes (Millipore, Billerica, MA, USA) using the Mini-PROTEAN Tetra Cell system (Bio-Rad, Hercules, CA, USA) at 100 V for 1 h. Membranes were blocked with 5% (w/v) skim milk in Tris-buffered saline containing 0.1% Tween-20 (TBST) for 1 h at room temperature, followed by overnight incubation at 4 °C with the following primary antibodies: p-AMPKα (1:1000, #2535S, Cell Signaling Technology, Danvers, MA, USA), AMPKα (1:1000, #2532, Cell Signaling Technology), Runx2 (1:1000, #12556S, Cell Signaling Technology), β-catenin (1:1000, ab32572, Abcam, Cambridge, UK), phospho-GSK3β (Ser9) (1:1000, #9323S, Cell Signaling Technology), GSK3β (1:1000, #9315S, Cell Signaling Technology), PPAR-γ (1:1000, #2443S, Cell Signaling Technology), ALP (1:500, PA5-106391, Thermo Fisher Scientific), α-tubulin (1:1000, MA5-116869, Thermo Fisher Scientific). After washing three times with TBST (10 min each), membranes were incubated with horseradish peroxidase (HRP)-conjugated secondary antibodies: anti-rabbit IgG (1:5000, #7074S, Cell Signaling Technology) or anti-mouse IgG (1:5000, #7076S, Cell Signaling Technology) for 45 min at room temperature. Immunoreactive bands were visualized using an enhanced chemiluminescence (ECL) detection kit (Pierce ECL, Thermo Fisher Scientific) and captured with a FUSION Solo imaging system (Vilber Lourmat, France). Band intensities were quantified using ImageJ software (NIH, Bethesda, MD, USA), and all protein expression levels were normalized to α-tubulin as the internal loading control.

### 4.10. Enzyme-Linked Immunosorbent Assay (ELISA)

Blood samples were collected immediately after euthanasia and allowed to clot at room temperature for 30 min. The samples were then centrifuged at 3000× *g* for 15 min at 4 °C, and the separated serum was stored at −80 °C until analysis.

Serum concentrations of bone turnover markers—including γ-carboxylated osteocalcin (Gla-OC), undercarboxylated osteocalcin (Glu-OC), bone-specific alkaline phosphatase (BALP), and calcium (Ca^2+^)—were quantified using commercially available enzyme-linked immunosorbent assay (ELISA) kits according to the manufacturers’ instructions: Gla-OC (MK127, Takara Bio Inc., Shiga, Japan), Glu-OC (MK129, Takara Bio Inc., Shiga, Japan), BALP (CSB-E11914m, Cusabio, Houston, TX, USA), and calcium (E-BC-K103-M, Elabscience, Houston, TX, USA). All measurements were performed in triplicate, and the mean values were used for statistical analysis.

### 4.11. Cell Culture and Cytotoxicity

The human osteoblast-like cell line MG63 (CVCL_0426; Korea Cell Line Bank, Seoul, Republic of Korea) and macrophages cell line RAW 264.7 were cultured in Dulbecco’s Modified Eagle Medium (DMEM; Gibco, Grand Island, NY, USA) supplemented with 10% fetal bovine serum (FBS; Gibco) and 1% antibiotic–antimycotic solution (100 U/mL penicillin, 100 µg/mL streptomycin, and 0.25 µg/mL amphotericin B; Gibco). Cells were maintained at 37 °C in a humidified incubator containing 5% CO_2_.

For osteoblastic induction, the culture medium was replaced every two days with differentiation medium containing 0.5 mM L-ascorbic acid and 10 mM β-glycerophosphate (AA/βGP) [40]. For osteoclastic induction, the culture medium was replaced every two days with differentiation medium containing 100 ng receptor activator of nuclear factor-κB ligand (RANKL).

To determine a non-cytotoxic concentration range of CGAC, cell viability was evaluated using the WST-8 assay (EZ-Cytox, DoGenBio, Seoul, Republic of Korea). MG63 and RAW 264.7 cells (1 × 10^5^ cells/well) were seeded in 96-well plates and allowed to adhere for 12 h before exposure to CGAC (25, 50, or 100 µg/mL) for 24 h. Following treatment, 10 µL of WST-8 reagent was added to each well and incubated for 2 h at 37 °C. Absorbance was measured at 450 nm using a microplate reader (Molecular Devices, Sunnyvale, CA, USA). Cell viability was expressed as a percentage relative to untreated control cells.

### 4.12. ALP Staining and Activity

MG63 cells (2 × 10^4^ cells/well) were seeded in 6-well plates and allowed to attach for 12 h. Cells were then treated with CGAC (25, 50, or 100 µg/mL) in osteogenic differentiation medium containing 0.5 mM L-ascorbic acid and 10 mM β-glycerophosphate. Treatments were refreshed every two days for 7 days. On day 7, ALP staining was performed using the BCIP/NBT Liquid Substrate System (Sigma-Aldrich, St. Louis, MO, USA). Briefly, cells were fixed with 10% neutral-buffered formalin for 20 min at room temperature, rinsed twice with distilled water, and incubated in BCIP/NBT solution for 3 h in the dark at room temperature. After washing three times with distilled water, ALP-positive cells were identified by blue-purple coloration under a light microscope (Axio Observer, Carl Zeiss, Jena, Germany). For the quantitative ALP activity assay, MG63 cells were cultured under the same differentiation conditions as those used for ALP staining but in separate 6-well plates to avoid interference from the staining procedure. After the 7-day differentiation period, cells were lysed, and ALP enzymatic activity was determined using the 1-Step™ p-Nitrophenyl Phosphate (pNPP) substrate solution (Thermo Fisher Scientific, Waltham, MA, USA) according to the manufacturer’s instructions. Absorbance was measured at 405 nm, and activity was normalized to total protein content.

### 4.13. Alizarin Red Staining and Activity

For mineralization assays, MG63 cells (2 × 10^4^ cells/well) were seeded in 6-well plates and allowed to attach for 12 h before treatment with CGAC (25, 50, or 100 µg/mL) in differentiation medium containing 0.5 mM L-ascorbic acid and 10 mM β-glycerophosphate. The treatment medium was renewed every two days for 14 or 21 days. At the indicated time points, calcium deposition was assessed using Alizarin Red S (ARS) staining (Sigma-Aldrich, MO, USA). Cells were fixed with 10% formalin for 20 min at room temperature, rinsed twice with distilled water, and incubated with 2% ARS solution (pH 4.2) for 2 h in the dark. After staining, cells were washed three times with distilled water to remove unbound dye. For quantitative analysis, bound ARS was eluted by incubation with 10% pyridinium chloride (Sigma-Aldrich) for 1 h, and the absorbance was measured at 550 nm using a microplate spectrophotometer (Molecular Devices). Results were expressed as relative mineralization compared to control cultures.

### 4.14. TRAP Staining and Activity

RAW 264.7 cells were plated in 96-well plates at 1 × 10^4^ cells/well and cultured. After 4 h, they were treated with CGAC (25, 50, or 100 µg/mL) in Alpha-MEM medium (Gibco, Grand Island, NY, USA) containing 100 ng/mL RANKL (PeproTech, New Jersey, USA). Treatments were refreshed every two days for 7 days. At the end of the differentiation period, cells were washed with PBS and stained using the TRAP (tartrate-resistant acid phosphatase) Staining Kit (Takara Bio Inc., Japan) according to the manufacturer’s instructions. TRAP-positive osteoclasts were identified as multinucleated cells (>3 nuclei) exhibiting red–purple cytoplasmic staining and were counted in at least five randomly selected fields per well. The TRAP-stained area was quantified using ImageJ software (NIH, Bethesda, MD, USA).

### 4.15. Statistical Analysis

All data are presented as mean ± standard deviation (SD). Prior to statistical testing, normality was assessed using the Shapiro–Wilk test and homogeneity of variance was assessed using the Brown–Forsythe test. For datasets satisfying these assumptions, group differences were analyzed using one-way ANOVA followed by Dunnett’s post hoc test. When normality assumptions were not met, the Kruskal–Wallis test followed by Dunn’s multiple-comparison test was used. For datasets with unequal variances, Welch’s ANOVA followed by an appropriate unequal-variance multiple-comparison test was applied. A two-sided *p*-value < 0.05 was considered statistically significant. Statistical analyses were conducted using GraphPad Prism 7 software (GraphPad, Inc., La Jolla, CA, USA).

## 5. Conclusions

In conclusion, CGAC attenuated bone loss and improved multiple indices of dysregulated bone remodeling in an ORX-induced mouse model. The observed effects were associated with reduced marrow adiposity, enhanced osteoblast-related activity, and suppression of osteoclastogenic responses, with possible involvement of AMPK- and Wnt/β-catenin-related signaling. Although these findings support the therapeutic potential of CGAC for androgen-deficiency-related bone loss, further studies are required to clarify the precise mechanisms, validate pathway-level effects, and establish long-term safety and translational relevance.

## Data Availability

The original contributions presented in the study are included in the article/Supplementary Materials; further inquiries can be directed to Eun-Jung Lee or Chang-Gue Son (corresponding author).

## Supplementary Materials

The following supporting information can be downloaded at: https://www.mdpi.com/article/10.3390/ph19040555/s1, Figure S1: Experimental design and toxicity of CGAC in vivo and in vitro; Table S1: Mean differences and 95% confidence intervals for the principal comparisons shown in Figure 2; Table S2: Mean differences and 95% confidence intervals for the principal comparisons shown in Figure 3; Table S3: Mean differences and 95% confidence intervals for the principal comparisons shown in Figure 4; Table S4: Mean differences and 95% confidence intervals for the principal comparisons shown in Figure 5; Table S5: Mean differences and 95% confidence intervals for the principal comparisons shown in Figure 6.

## Abbreviations

The following abbreviations are used in this manuscript:

ARS	Alizarin red staining
ALP	Alkaline phosphatase
AMPKα	AMP-activated protein kinase alpha
ADT	Androgen deprivation therapy
AA/βGP	Ascorbic acid and β-glycerophosphate
BALP	Bone-specific alkaline phosphatase
BCA	Bicinchoninic acid
AV/TV	Bone marrow adipocyte volume/tissue volume
BMD	Bone mineral density
BV/TV	Bone volume/total volume
ECL	Enhanced chemiluminescence
ELISA	Enzyme-Linked Immunosorbent Assay
FDA	Food and Drug Administration
H&E	Hematoxylin and eosin
HPLC	High-performance liquid chromatography
OC	Osteocalcin
ORX	Orchiectomy
OVX	Ovariectomy
Ob.S/BS	Osteoblast surfaces/bone surface
p-AMPKα	Phospho-AMP-activated protein kinase alpha
PPAR-γ	Peroxisome proliferator-activated receptor gamma
Runx2	Runt-related transcription factor 2
SDs	Standard deviations
TEMED T	Tetraethyl ethylenediamine
PVDF	Polyvinylidene fluoride
Tb.N	Trabecular bone number
Tb.Th	Trabecular bone thickness
µCT	Micro-computed tomography
