# Supplementary material for: A Standard Herbal Formula, CGAC, Attenuates Bone Loss by Normalizing Low-Bone Turnover Stagnation in an Orchiectomy-Induced Mouse Model
Source: Pharmaceuticals (Basel). 2026 Mar 31;19(4):555. doi: 10.3390/ph19040555 (PMC13118365; doi:10.3390/ph19040555)
Supplement: Supplementary file 1 [file pharmaceuticals-19-00555-s001.zip › Supplementary Figure.pdf]

Supplementary Figure S1

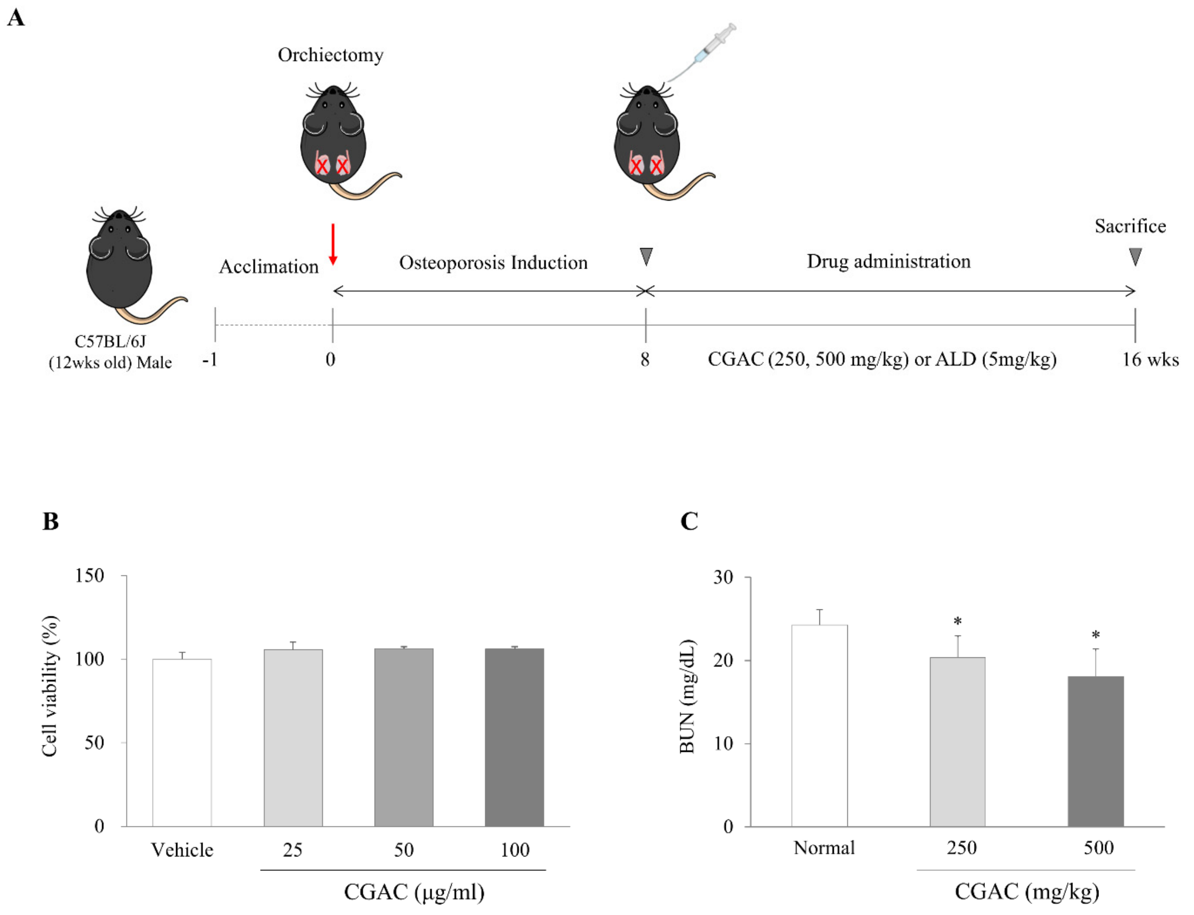

Supplementary Figure S1. Experimenal design and toxicity of CGAC In vivo and In vitro. A synthetic scheme presents the information regarding experimental design (A). The cytotoxicity of CGAC (25, 50, and 100 μg/mL) in the MG63 cells (B) and serum levels of BUN in CGAC treated mice (C).
