# Supplementary material for: A Standard Herbal Formula, CGAC, Attenuates Bone Loss by Normalizing Low-Bone Turnover Stagnation in an Orchiectomy-Induced Mouse Model
Source: Pharmaceuticals (Basel). 2026 Mar 31;19(4):555. doi: 10.3390/ph19040555 (PMC13118365; doi:10.3390/ph19040555)
Supplement: Supplementary file 1 [file pharmaceuticals-19-00555-s001.zip › Supplementary Table.pdf]

Table S1. Mean differences and 95% confidence intervals for the principal comparisons shown in Figure. 2.

Figure. No.	Experiment	Dunnett's multiple comparisons test	Mean Diff.	95.00% CI of diff.
Figure. 2	μCT BMD	ORX vs. Sham	-0.0369	-0.04734 to -0.02646
		ORX vs. 250	-0.01743	-0.02387 to -0.002990
		ORX vs. 500	-0.01762	-0.02806 to -0.007181
		ORX vs. ALD	-0.01379	-0.02423 to -0.003351
	BV/TV	ORX vs. Sham	-2.913	-3.615 to -2.211
		ORX vs. 250	-0.9914	-1.693 to -0.2894
		ORX vs. 500	-0.7601	-1.362 to -0.04192
		ORX vs. ALD	-0.7894	-1.491 to -0.08743
	Tb.Th	ORX vs. Sham	-0.007079	-0.01278 to -0.001376
		ORX vs. 250	-0.007207	-0.01291 to -0.001504
		ORX vs. 500	-0.008164	-0.01387 to -0.002461
		ORX vs. ALD	-0.004785	-0.01049 to 0.0009176
	Tb.N	ORX vs. Sham	-0.5106	-0.6500 to -0.3711
		ORX vs. 250	-0.1444	-0.2438 to -0.003508
		ORX vs. 500	-0.1552	-0.2380 to -0.004093
		ORX vs. ALD	-0.1455	-0.2749 to 0.003992
	SMI	ORX vs. Sham	0.8096	0.5357 to 1.084
		ORX vs. 250	0.5239	0.2500 to 0.7978
		ORX vs. 500	0.6713	0.3974 to 0.9452
		ORX vs. ALD	0.5135	0.2396 to 0.7874

Table S2. Mean differences and 95% confidence intervals for the principal comparisons shown in Figure. 3.

Figure. No.	Experiment	Dunnett's multiple comparisons test	Mean Diff.	95.00% CI of diff.
Figure. 3	DXA BMD on the final day	ORX vs. Sham	-0.01543	-0.01770 to -0.01317
		ORX vs. 250	-0.005023	-0.007399 to -0.002647
		ORX vs. 500	-0.005743	-0.008119 to -0.003367
		ORX vs. ALD	-0.004343	-0.006719 to -0.001967
	BMC (%)	ORX vs. Sham	-0.3582	-0.6510 to -0.06536
		ORX vs. 250	-0.4562	-0.7490 to -0.1634
		ORX vs. 500	-0.41	-0.7028 to -0.1172
		ORX vs. ALD	-0.6067	-0.8995 to -0.3139
	BALP (ng/ml)	ORX vs. Sham	-26.04	-35.12 to -16.96
		ORX vs. 250	-41.76	-50.84 to -32.68
		ORX vs. 500	-33.1	-42.18 to -24.02
		ORX vs. ALD	-52.44	-61.52 to -43.36
	CTX-1 (ng/ml)	ORX vs. Sham	-1327	-1390 to -1264
		ORX vs. 250	-1406	-1469 to -1343
		ORX vs. 500	-1593	-1656 to -1529
		ORX vs. ALD	-1795	-1858 to -1732
	Gla/Glu-OC (ng/ml)	ORX vs. Sham	-54.66	-63.09 to -46.22
		ORX vs. 250	-10.91	-19.34 to -2.473
		ORX vs. 500	-11.26	-19.69 to -2.824
		ORX vs. ALD	4.829	-3.604 to 13.26
	Calcium (mmol/L)	ORX vs. Sham	1.433	1.260 to 1.607
		ORX vs. 250	0.4533	0.2795 to 0.6271
		ORX vs. 500	0.9233	0.7495 to 1.097
		ORX vs. ALD	0.5333	0.3595 to 0.7071

Table S3. Mean differences and 95% confidence intervals for the principal comparisons shown in Figure. 4.

Figure. No.	Experiment	Dunnett's multiple comparisons test	Mean Diff.	95.00% CI of diff.
Figure. 4	AV/TV (%)	ORX vs. Sham	54.35	51.87 to 56.83
		ORX vs. 250	28.06	25.58 to 30.54
		ORX vs. 500	25.45	22.97 to 27.93
		ORX vs. ALD	44.59	42.11 to 47.07
	Ob.S/BS (%)	ORX vs. Sham	-24	-26.67 to -21.33
		ORX vs. 250	-15	-17.67 to -12.33
		ORX vs. 500	-21.05	-23.72 to -18.38
		ORX vs. ALD	-17	-19.67 to -14.33
	Oc.S/BS (%)	ORX vs. Sham	-54.35	-56.83 to -51.87
		ORX vs. 250	-28.9	-31.38 to -26.42
		ORX vs. 500	-26.29	-28.77 to -23.81
		ORX vs. ALD	-9.758	-12.24 to -7.278
	p-AMPK	ORX vs. Sham	-1.231	-1.465 to -0.9980
		ORX vs. 250	-1.29	-1.523 to -1.057
		ORX vs. 500	-0.944	-1.177 to -0.7105
		ORX vs. ALD	-0.5861	-0.8195 to -0.3526
	Runx2	ORX vs. Sham	-0.3592	-0.5015 to -0.2169
		ORX vs. 250	-0.2263	-0.3686 to -0.08401
		ORX vs. 500	-0.209	-0.3513 to -0.06675
		ORX vs. ALD	0.1231	-0.01920 to 0.2654
	PPAR-γ	ORX vs. Sham	0.7295	0.5618 to 0.8973
		ORX vs. 250	0.8695	0.7018 to 1.037
		ORX vs. 500	0.6764	0.5087 to 0.8441
		ORX vs. ALD	0.8448	0.6770 to 1.012

Table S4. Mean differences and 95% confidence intervals for the principal comparisons shown in Figure. 5.

Figure. No.	Experiment	Dunnett's multiple comparisons test	Mean Diff.	95.00% CI of diff.
Figure. 5	ALP staining D7	AA+βGP vs. Veh	0.6	0.3379 to 0.8621
		AA+βGP vs. 25	-0.31	-0.5721 to -0.04790
		AA+βGP vs. 50	-0.32	-0.5821 to -0.05790
		AA+βGP vs. 100	-1.6	-1.862 to -1.338
	ALP staining D14	AA+βGP vs. Veh	0.574	0.3907 to 0.7573
		AA+βGP vs. 25	-0.1236	-0.3069 to 0.05974
		AA+βGP vs. 50	-0.2156	-0.3989 to -0.03226
		AA+βGP vs. 100	-1.12	-1.303 to -0.9367
	ALP staining D21	AA+βGP vs. Veh	0.675	0.4665 to 0.8835
		AA+βGP vs. 25	-0.38	-0.5885 to -0.1715
		AA+βGP vs. 50	-0.56	-0.7685 to -0.3515
		AA+βGP vs. 100	-1.32	-1.529 to -1.111
	Area of osteoclasts (%)	RANKL vs. Vehicle	91.33	82.80 to 99.86
		RANKL vs. 25	21.33	12.80 to 29.86
		RANKL vs. 50	44.33	35.80 to 52.86
		RANKL vs. 100	74.33	65.80 to 82.86
	No. of TRAP + cells/well	RANKL vs. Vehicle	236	190.9 to 281.1
		RANKL vs. 25	15.33	-29.80 to 60.47
		RANKL vs. 50	80	34.86 to 125.1
		RANKL vs. 100	162.3	117.2 to 207.5

Table S5. Mean differences and 95% confidence intervals for the principal comparisons shown in Figure. 6.

Figure. No.	Experiment	Dunnett's multiple comparisons test	Mean Diff.	95.00% CI of diff.
Figure. 6	p-GSK3β	AA/β-GP vs. Vehicle	-0.2314	-0.003475 to -0.2662
		AA/β-GP vs. 25	-0.1485	-0.2834 to -0.01371
		AA/β-GP vs. 50	-0.3164	-0.4512 to -0.1815
		AA/β-GP vs. 100	-0.3458	-0.4806 to -0.2109
	β-catenin	AA/β-GP vs. Vehicle	0.1386	0.02722 to 0.2500
		AA/β-GP vs. 25	0.09856	-0.01283 to 0.2099
		AA/β-GP vs. 50	-0.1539	-0.2653 to -0.04252
		AA/β-GP vs. 100	-0.1504	-0.2618 to -0.03903
	Runx2	AA/β-GP vs. Vehicle	0.2866	0.1441 to 0.4292
		AA/β-GP vs. 25	0.07776	-0.06479 to 0.2203
		AA/β-GP vs. 50	-0.2319	-0.3744 to -0.08934
		AA/β-GP vs. 100	-0.4118	-0.5544 to -0.2693
	p-GSK3β	Vehicle vs. AA/β-GP	-0.7068	-0.9987 to -0.4149
		Vehicle vs. 100	-1.533	-1.825 to -1.242
		Vehicle vs. Sclerostin_vehicle	0.1845	-0.1074 to 0.4763
		Vehicle vs. Sclerostin_AA/β-GP	0.1977	-0.09418 to 0.4896
		Vehicle vs. Sclerostin_100	0.2355	-0.05640 to 0.5273
	β-catenin	Vehicle vs. AA/β-GP	-0.6553	-0.9522 to -0.3584
		Vehicle vs. 100	-0.8294	-1.126 to -0.5325
		Vehicle vs. Sclerostin_vehicle	-0.7433	-1.040 to -0.4464
		Vehicle vs. Sclerostin_AA/β-GP	-1.283	-1.580 to -0.9858
		Vehicle vs. Sclerostin_100	-0.1604	-0.4573 to 0.1365
	Runx2	Vehicle vs. AA/β-GP	-0.286	-0.5004 to -0.07164
		Vehicle vs. 100	-0.4208	-0.6352 to -0.2064
		Vehicle vs. Sclerostin_vehicle	0.113	-0.1014 to 0.3274
		Vehicle vs. Sclerostin_AA/β-GP	0.4185	0.2041 to 0.6329
		Vehicle vs. Sclerostin_100	0.3789	0.1645 to 0.5933
